# Supplementary material for: Factors Associated with Uptake of Routine Measles-Containing Vaccine Doses among Young Children, Oromia Regional State, Ethiopia, 2021
Source: Vaccines (Basel). 2024 Jul 11;12(7):762. doi: 10.3390/vaccines12070762 (PMC11281473; doi:10.3390/vaccines12070762)
Supplement: Supplementary file 1 [file vaccines-12-00762-s001.zip › vaccines-3008443-supplementary.pdf]

Supplementary Materials

Table S1: Bivariate and multivariate association between caregiver, household, and child demographic factors and first dose of measles containing vaccine (MCV1) vaccination among children 12-23 months by settlement in Oromia Region, Ethiopia, N=598

**A. Rural Settlement, n=299**								
Characteristic	N	MCV1=Yes	Bivariate			Multivariate		
			OR	95% CI	p-value	aOR	95% CI	p-value
Caregiver's highest level of education completed					0.003			0.007
No formal education	140	71	Ref			Ref		
Primary	115	77	1.97	1.19, 3.30		2.03	1.10, 3.80	
Secondary	36	29	4.03	1.74, 10.5		4.35	1.62, 12.8	
Tertiary	8	5	1.62	0.38, 8.14		3.72	0.74, 20.7	
Caregiver's age in years					0.92			
18 to 26 years	175	108	Ref					
27 to 80 years	121	74	0.98	0.61, 1.58				
Number of children under 59 months living in household					0.38			
One	174	111	Ref					
Two	108	62	0.76	0.47, 1.25				
Three or four	16	8	0.57	0.20, 1.61				
Sex of child					0.45			
Male	130	76	Ref					
Female	169	106	1.20	0.75, 1.91				
Delivery location					<0.001			
Home	97	44	Ref					
At HF or on the way to HF	202	138	2.60	1.58, 4.29				
Caregiver believes that child has received all recommended vaccines					<0.001			<0.001
No/Don't remember	137	44	Ref			Ref		
Yes	161	138	12.7	7.29, 22.8		13.3	7.52, 24.5	
Number of vaccination visits child needs					0.008			
0-5 Visits	237	144	Ref					
6 visits (correct as per EPI schedule)	23	20	4.31	1.43, 18.6				
Named measles as a VPD					0.18			
No	41	21	Ref					
Yes	258	161	1.58	0.81, 3.07				
Heard of immunization against measles					0.012			
No	51	23	Ref					
Yes	248	159	2.17	1.18, 4.03				
Number of doses of measles vaccine that child is supposed to get								

Never heard of measles vaccine or don't know number of doses	185	100	Ref		0.003			
Heard of measles vaccine – One dose	52	35	1.75	0.93, 3.41				
Heard of measles vaccine – Two doses	58	45	2.94	1.52, 6.02				
Know of a family or community member who had measles					0.010			
No	213	120	Ref					
Yes	86	62	2.00	1.17, 3.50				
In the household, who makes the decision to immunize child?					<0.001			
Mother or father only (one parent)	86	39	Ref					
Both father and mother	207	140	2.52	1.51, 4.23				
Ever been sent home from health center due to vaccine stock-out?					0.020			
No	257	163	Ref					
Yes	39	17	0.45	0.22, 0.88				
Type of vaccination services available to your child					0.80			
Health facility (fixed)	201	125	Ref					
Outreach site	9	5	0.76	0.20, 3.15				
Both	87	51	0.86	0.52, 1.44				
Frequency of vaccination availability					0.64			
Every month	186	121	Ref					
Every week	74	49	1.05	0.60, 1.88				
Every day	1	1						
Walking time to vaccination center					0.030			
Between 1 to 6 hours	20	7	Ref					
30 mins to 1 hour	42	23	2.25	0.76, 7.06				
30 mins or less	233	149	3.29	1.30, 9.07				
How long do you wait at the vaccination center before the child is vaccinated?								
Between 1 to 6 hours	43	25	Ref		0.50			
30 mins to 1 hour	71	41	0.98	0.45, 2.12				
30 mins or less	176	114	1.32	0.66, 2.60				

B. Urban Settlement, n=299

Characteristic	Bivariate					Multivariate		
	N	MCV1=Yes	OR	95% CI	p-value	aOR	95% CI	p-value
Caregiver's[1] highest level of education completed					<0.001			
No formal education	31	17	Ref					

Primary	95	72	2.58	1.10, 6.06				
Secondary	85	72	4.56	1.83, 11.7				
Tertiary	88	80	8.24	3.06, 23.7				
Caregiver's age in years					0.021			0.010
18 to 26 years	155	117	Ref			Ref		
27 to 80 years	143	123	2.00	1.11, 3.69		2.90	1.29, 6.93	
Number of children under 59 months living in household					<0.001			<0.001
One	209	183	Ref					
Two	80	54	0.30	0.16, 0.55		0.25	0.11,0.56	
Three or four	10	4	0.09	0.02, 0.35		0.09	0.01, 0.51	
Sex of child					0.33			
Male	146	121	Ref					
Female	153	120	0.75	0.42, 1.34				
Delivery location					0.047			
Home	18	11	Ref					
At HF or on the way to HF	281	130	2.87	1.01, 7.65				
Caregiver believes that child has received all recommended vaccines					<0.001			<0.001
No/Don't remember	105	64	Ref			Ref		
Yes	194	117	6.67	3.60, 12.8		4.55	2.17, 9.91	
Number of vaccination visits child needs					0.001			0.018
0-5 Visits	212	163	Ref			Ref		
6 visits (correct as per EPI schedule)	53	50	5.01	1.74, 21.2		4.43	1.26, 23.1	
Named measles as a VPD					<0.001			0.005
No	55	34	Ref			Ref		
Yes	244	207	3.46	1.80, 6.59		3.75	1.49, 9.60	
Heard of immunization against measles					0.21			
No	41	30	Ref					
Yes	258	211	1.65	0.74, 3.44				
Number of doses of measles vaccine that child is supposed to get					0.11			
Never heard of measles vaccine or don't know number of doses	162	124	Ref					
Heard of measles vaccine – One dose	40	32	1.23	0.54, 3.06				
Heard of measles vaccine – Two doses	93	81	2.07	1.05, 4.35				
Know of a family or community member who had measles					0.38			
No	215	176	Ref					
Yes	84	65	0.76	0.41, 1.43				

Characteristic	N	MCV =Yes	OR	95% CI	p-value	aOR	95% CI	p-value
In the household, who makes the decision to immunize child?					0.12			
Mother or father only (one parent)	47	34	Ref					
Both father and mother	249	205	1.78	0.85, 3.59				
Ever been sent home from health center due to vaccine stock-out?					<0.001			0.023
No	257	218	Ref			Ref		
Yes	41	23	0.23	0.11, 0.47		0.33	0.13, 0.86	
Type of vaccination services available to your child					<0.001			
Health facility (fixed)	202	218						
Outreach site	4	0						
Both	92	66	0.41	0.22, 0.75				
Frequency of vaccination availability					0.17			
Every month	117	102	Ref					
Every week	97	78	0.60	0.28, 1.26				
Every day	55	50	1.47	0.54, 4.73				
Walking time to vaccination center								
Between 1 to 6 hours	19	18	Ref		0.17			
30 mins to 1 hour	43	33	0.18	0.01, 1.07				
30 mins or less	228	184	0.23	0.01, 1.17				
How long do you wait at the vaccination center before the child is vaccinated?					0.58			
Between 1 to 6 hours	86	69	Ref					
30 mins to 1 hour	74	57	0.83	0.39, 1.77				
30 mins or less	135	112	1.20	0.59, 2.40				

N: Total number of surveyed children, MCV: Measles-containing vaccine, OR: Odds Ratio, aOR: Adjusted odds ratio, CI: Confidence interval, HF: Health Facility, EPI: Expanded program on vaccination, VPD: Vaccine Preventable Disease. All independent variables with p-value <0.15 in the bivariate analysis were added in the initial multivariate regression model

Table S2: Bivariate and multivariate association between caregiver, household, and child demographic characteristics, caregiver's knowledge, attitude, Practice and awareness factors and second dose of measles-containing vaccine (MCV2) vaccination among children 18-35 months by Settlement in Oromia Region, Ethiopia (N=**572**)

**A. Rural Settlement, n= 244**								
Characteristic	N	MCV2	Bivariate			Multivariate		
		=Yes	OR	95% CI	p-value	aOR	95% CI	p-value
Caregiver's highest level of education completed								
No formal education	122	41	Ref		0.13			
Primary	82	34	1.40	0.78, 2.50				
Secondary	32	18	2.54	1.15, 5.70				
Tertiary	8	3	1.19	0.23, 5.07				

Caregiver's age in years					0.035			
18 to 26 years	117	38	Ref					
27 to 80 years	127	58	1.75	1.04, 2.96				
Number of children under 59 months living in household					0.53			
One	154	62						
Two	80	32	0.99	0.57, 1.71				
Three or four	9	2	0.42	0.06, 1.82				
Sex of child			Ref		0.30			
Male	104	37						
Female	140	59	1.32	0.78, 2.24				
Delivery location					0.080			
Home	63	19						
At HF or on the way to HF	181	77	1.71	0.94, 3.22				
Caregiver believes that child has received all recommended vaccines					<0.001			<0.001
No/Don't remember	59	6	Ref			Ref		
Yes	184	89	8.28	3.64, 22.4		10.7	3.93, 38.1	
Number of vaccination visits child needs					<0.001			0.004
0-5 visits	186	68	Ref			Ref		
6 visits (correct as per EPI schedule)	29	22	5.45	2.32, 14.4		3.98	1.54, 11.6	
Named measles as a VPD					0.025			
No	32	7	Ref					
Yes	212	89	2.58	1.12, 6.71				
Heard of immunization against measles					<0.001			
No	35	5	Ref					
Yes	209	91	4.63	1.87, 14.0				
Number of doses of measles vaccine that child is supposed to get					0.002			0.027
Never heard of measles vaccine or don't know # of doses	135	46	Ref			Ref		
Heard of measles vaccine One dose	39	11	0.76	0.34, 1.63		0.47	0.17, 1.17	
Heard of measles vaccine Two doses	68	39	2.6	1.44, 4.77		1.77	0.88, 3.63	
Know of a family or community member who had measles					0.027			
No	153	32	Ref					
Yes	91	44	1.82	1.07, 3.10				
In the household, who makes the decision to immunize child?					0.018			
Mother or father only (one parent)	70	19	Ref					
Both father and mother	171	74	2.05	1.13, 3.83				
Ever been sent home from health center due to vaccine stock-out?					0.68			
No	214	83	Ref					
Yes	28	12	1.18	0.52, 2.62				
Type of vaccination services available to your child					0.84			
Health facility (fixed)	180	69						
Outreach site	2	1	1.61	0.06, 41.1				

Both	62	26	1.16	0.64, 2.09				
Frequency of vaccination availability					0.77			
Every month	163	69						
Every week	50	24	1.26	0.66, 2.38				
Every day	2	1	1.36	0.05, 34.9				
Walking time to vaccination center					0.024			0.023
Between 1 to 6 hours	18	2	Ref			Ref		
30 mins to 1 hour	39	15	5.00	1.19, 34.5		11.3	1.73, 2.27	
30 mins or less	181	75	5.66	1.55, 36.5		9.08	1.63, 1.71	
How long do you wait at the vaccination center before the child is vaccinated?					0.093			
Between 1 to 6 hours	25	12						
30 mins to 1 hour	63	18	0.43	0.16, 1.13				
30 mins or less	154	66	0.81	0.35, 1.92				

B. Urban Settlement, n= 328

Characteristic			Bivariate			Multivariate		
	N	MCV2 =Yes	OR	95% CI	p-value	aOR	95% CI	p-value
Caregiver's[1] highest level of education completed					0.034			
No formal education	33	13	Ref					
Primary	114	59	1.65	0.76, 3.70				
Secondary	87	58	3.08	1.36, 7.19				
Tertiary	94	54	2.08	0.93, 4.7				
Caregiver's age in years					0.67			
18 to 26 years	161	92						
27 to 80 years	166	91	0.91	0.59, 1.41				
Number of children under 59 months living in household					0.013			0.018
One	232	134	Ref			Ref		
Two	89	48	0.86	0.52, 1.40		0.67	0.38, 1.20	
Three or four	5	0	0			0.00		
Sex of child					0.13			
Male	169	88						
Female	15	98	1.40	0.91, 2.18				
Delivery location					0.10			
Home	5	1						
At HF or on the way to HF	323	183	5.23	0.76, 103				
Caregiver believes that child has received all recommended vaccines					<0.001			<0.001
No/Don't remember	63	10	Ref			Ref		
Yes	265	174	10.1	5.12, 22.0		9.1	4.23, 22.2	
Number of vaccination visits child needs					<0.001			0.002
0-5	209	106	Ref			Ref		
6 (correct as per EPI schedule)	79	61	3.29	1.85, 6.09		2.65	1.43, 5.08	
Named measles as a VPD					0.35			
No	50	25						
Yes	278	159	1.34	0.73, 2.45				
Heard of immunization against measles					0.023			
No	28	10	Ref					
Yes	300	174	2.49	1.13, 5.77				

Number of doses of measles vaccine that child is supposed to get					0.004
Never heard of measles vaccine or don't know # of doses	156	73	Ref		
Heard of measles vaccine One dose	38	21	1.4	0.69, 2.89	
Heard of measles vaccine Two doses	130	86	2.22	1.38, 3.61	
Know of a family or community member who had measles					>0.99
No	230	129	Ref		
Yes	98	55	1.00	0.62, 1.62	
In the household, who makes the decision to immunize child?					0.086
Mother or father only (one parent)	51	23			
Both father and mother	277	161	1.69	0.93, 3.10	
Ever been sent home from health center due to vaccine stock-out?					0.026
No	280	165	Ref		
Yes	44	18	0.48	0.25, 0.92	
Type of vaccination services available to your child					0.42
Health facility (fixed)	240	137			
Both	86	45	0.83	0.50, 1.36	
Frequency of vaccination availability					0.97
Every month	128	73	Ref		
Every week	107	60	0.96	0.57, 1.62	
Every day	69	40	1.04	0.58, 1.89	
Walking time to vaccination center					0.005
Between 1 to 6 hours	29	24	Ref		
30 mins to 1 hour	42	22	0.23	0.07, 0.68	
30 mins or less	249	131	0.23	0.08, 0.58	
How long do you wait at the vaccination center before the child is vaccinated?					0.65
Between 1 to 6 hours	91	52	Ref		
30 mins to 1 hour	82	49	1.11	0.61, 2.05	
30 mins or less	151	81	0.87	0.51, 1.46	

N: Total number of surveyed children, MCV: Measles-containing vaccine, OR: Odds Ratio, aOR: Adjusted odds ratio, CI: Confidence interval, HF: Health Facility, EPI: Expanded program on vaccination, VPD: Vaccine Preventable Disease. All independent variables with p-value <0.15 in the bivariate analysis were added in the initial multivariate regression model
